# Surgical education in the post-COVID era: an EAES DELPHI-study

**DOI:** 10.1007/s00464-022-09762-1

**Published:** 2022-11-30

**Authors:** Tim M. Feenstra, Patricia Tejedor, Dorin E. Popa, Nader Francis, Marlies P. Schijven

**Affiliations:** 1 Amsterdam UMC Location University of Amsterdam, Surgery, Meibergdreef 9, Amsterdam, The Netherlands; 2 Amsterdam Gastroenterology and Metabolism, Amsterdam, The Netherlands; 3 Amsterdam Public Health, Digital Health, Amsterdam, The Netherlands; 4 Department of Colorectal Surgery, University Hospital Gregorio Marañón, Madrid, Spain; 5 Department of General Surgery, Linköping University Hospital, 581 85 Linköping, Sweden; 6 Department of Surgery, Yeovil District Hospital, Higher Kingston, Yeovil, BA21 4AT UK; 7 The Griffin Institute, Northwick Park and St Mark’s Hospital, Y Block, Watford Rd, Harrow, HA1 3UJ UK

**Keywords:** Training, COVID-19, Delphi, Consensus, Education, Surgery, Laparoscopy, Laparoscopic surgery

## Abstract

**Backgrounds:**

To date, it is unclear what the educational response to the restrictions on minimally invasive surgery imposed by the COVID-19 pandemic have been, and how MIS-surgeons see the post-pandemic future of surgical education. Using a modified Delphi-methodology, this study aims to assess the effects of COVID on MIS-training and to develop a consensus on the educational response to the pandemic.

**Methods:**

A three-part Delphi study was performed among the membership of the European Association of Endoscopic Surgery (EAES). The first survey aimed to survey participants on the educational response in four educational components: training in the operating room (OR), wet lab and dry lab training, assessment and accreditation, and use of digital resources. The second and third survey aimed to formulate and achieve consensus on statements on, and resources in, response to the pandemic and in post-pandemic MIS surgery.

**Results:**

Over 247 EAES members participated in the three rounds of this Delphi survey. MIS-training decreased by 35.6–55.6%, alternatives were introduced in 14.7–32.2% of respondents, and these alternatives compensated for 32.2–43.2% of missed training. OR-training and assessments were most often affected due to the cancellation of elective cases (80.7%, and 73.8% affected, respectively). Consensus was achieved on 13 statements. Although digital resources were deemed valuable alternatives for OR-training and skills assessments, face-to-face resources were preferred. Videos and hands-on training–wet labs, dry labs, and virtual reality (VR) simulation–were the best appreciated resources.

**Conclusions:**

COVID-19 has severely affected surgical training opportunities for minimally invasive surgery. Face-to-face training remains the preferred training method, although digital and remote training resources are believed to be valuable additions to the training palette. Organizations such as the EAES are encouraged to support surgical educators in implementing these resources. Insights from this Delphi can guide (inter)national governing training bodies and hospitals in shaping surgical resident curricula in post pandemic times.

The COVID-19 pandemic has severely disrupted surgical care. Healthcare professionals are quarantined, surgeries are postponed or cancelled to reduce stress on hospital capacity, and professionals and resources are reallocated to COVID care [1–4]. While this has postponed care affecting many patients, it has undoubtedly also impacted surgical education and training in all fields of surgery, including Minimally Invasive Surgery (MIS). Due to the pandemic, surgical residents’ exposure to MIS has been markedly restricted, MIS training centres had to close, and all educational activities were scaled down in favour of clinical COVID-related care [5–7]. This resulted in a significant decline of residents’ clinical activity, and residents felt that their surgical skills training had suffered during the pandemic [7]. To safeguard the proficiency of future minimally invasive surgeons, surgical educators needed to adapt and be creative. Traditional physical training methods in the OR, in labs or skills centres are believed to be insufficient because of their or limited availability–if they are available at all. Hence there is a need to introduce alternate training resources making use of digital technologies that may provide training at a distance. Through these adaptations and augmentations, the COVID-19 pandemic may have acted as a catalyst inflicting change of workflow and enforcing rapid change management.

To improve and futureproof the post-COVID MIS-curriculum, it is essential that training resources are thoroughly evaluated and insights are shared. This three-round modified Delphi aims to assess the effects of COVID on physical and digital training provision and to develop a consensus on the educational response to the pandemic–by surveying the experiences and expectations of the membership of the European Association of Endoscopic Surgery (EAES). The objectives of this study are: (1) To canvas the EAES membership on the impact of COVID on surgical resident training, (2) To identify current surgical educational issues, gaps, and the role of digital platforms to support surgical education, (3) To formulate statements of recommendation on the educational response to the pandemic, and (4) To achieve consensus among the members on resources and statements.

## Methods

This study was conducted through a web-based modified Delphi survey between December 2020 and July 2021. Due to the non-invasive nature of this study and the anonymous nature of collected survey data IRB approval was not needed. To ensure applicability and support a steering group (SG) was formed consisting two coordinating researchers: TF and PT, supported by members of several EAES committees: DP of the educational committee, NF from the research committee, and MS of the technological committee. The SG developed and screened the survey items and established the procedure.

All surveys were constructed using SurveyMonkey (SurveyMonkey Inc., San Mateo, California, USA, www.surveymonkey.com) and distributed by the EAES executive office to the EAES membership. An e-mail containing a link to the survey was sent out for each round of the surveys. Participation was voluntary, no compensation was offered and all responses were anonymized. The aim of the Delphi and an explanation of each of the surveys was provided at the start of each Delphi. Consent for participation and use of the data was implied by completion of the survey. Each round of the survey was open for one month, with a reminder sent after 2 weeks.

The Delphi process was divided in two phases, totalling three survey rounds. In phase one, one survey was sent out which was aimed at surveying the members on the educational response to the pandemic in four areas. (1) resident training in the operating room (OR), (2) resident training wet labs and dry labs, (3) assessments and skills accreditations of residents, and (4) the implementation and use of digital resources in surgical resident training. Participants were canvassed on the impact of COVID on surgical care and education, and asked to identify which adaptations were made and which digital resources were introduced in the educational response to the pandemic. Based on these results statements were formulated by the SG which were used in phase two of the study–which was aimed at reaching consensus on the educational response by ranking the importance of the resources and statements.

During phase two, two surveys were sent out. First, participants were asked to rank the importance of statements or usefulness of resources based on a five-point Likert-scale–ranging from very unimportant/very un-useful to very important/very useful. Consensus was achieved when ≥ 70% of the participants agreed on importance or usefulness. When consensus was achieved, participants were asked to rank the resources and statements based on their importance/usefulness for day to day practice in the third survey. After consensus and ranking of the statements and resources, the SG discussed the statements and formed them into recommendations. In accordance with Delphi-methodology free text options were provided wherever possible. All suitable questions in the surveys contained an answer option “Other”, which opened a free text box and all surveys contained an opportunity to ask questions and/or provide feedback at the end of the survey. Additionally, because the aim of the first survey was to canvas the EAES membership, participants were requested to elaborate on their answers in free text boxes.

Statistical analyses were performed using the Statistical Package for the Social Sciences (SPSS, IBM Corp. Released 2016. IBM SPSS Statistics for Windows, Version 24.0. Armonk, NY: IBM Corp.). Data are presented as number of cases and their percentages and means with standard deviation (SD) for the characteristics of participants. Data on statements and resources is presented as percentage of agreement, followed by the mean rank score. The mean rank score ranges from the highest possible rank (one) to the lowest rank, which equals the total number of resources or statements that was voted on. Hence, a lower score indicates higher preference by respondents.

## Results

The number of participants varied from 247 to 317 per survey, and included a wide range of countries and subspecialties. As depicted in Table 1 and in concordance with the EAES membership, the mean age in the surveys was 47 ± 11 years old, a small a minority (15–17.5%) of respondents was female, and a large consistent majority of respondents held a senior function in hospital–consultant, professor, director, or head of the department (75.1–87.1%).

**Table 1:** Characteristics of survey respondents per survey

	Round 1 (*n* = 273)		Round 2 (*n* = 317)		Round 3 (*n* = 247)	
Top contributing countries						
Italy	54	19.8%	76	24.0%	38	15.4%
Spain	22	8.1%	30	9.5%	21	8.5%
Romania	18	6.6%	22	6.9%	18	7.3%
UK	18	6.6%	14	4.4%	15	6.1%
Greece	14	5.1%	24	7.6%	23	9.3%
Netherlands	13	4.8%	13	4.1%	9	3.6%
Switzerland	10	3.7%	7	2.2%	5	2.0%
Japan	9	3.3%	3	0.9%	15	6.1%
Germany	7	2.6%	9	2.8%	10	4.0%
India	6	2.2%	4	1.3%	8	3.2%
Poland	5	1.8%	10	3.2%	4	1.6%
France	5	1.8%	9	2.8%	3	1.2%
Austria	3	0.7%	7	2.2%	2	0.8%
Sweden	2	0.7%	6	1.9%	6	2.4%
Age (mean ± SD)	47	± 11	47	± 11	47	± 11
Gender: Female	41	15.0%	50	16.0%	43	17.5%
Position						
Resident	28	10.3%	22	6.9%	16	6.5%
Fellow	24	8.8%	14	4.4%	21	8.5%
Senior (consultant, professor, director, department head)	205	75.1%	276	87.1%	208	84.2%
Type of surgery						
General Surgery	212	77.7%	245	77.3%	176	71.3%
Upper GI	106	38.8%	120	37.9%	98	39.7%
Bariatric and/or reflux	73	26.7%	84	26.5%	61	24.7%
HPB	66	24.2%	72	22.7%	57	23.1%
Colorectal	154	56.4%	194	61.2%	139	56.3%
Endocrine	40	14.7%	42	13.2%	33	13.4%
Urology	2	0.7%	3	0.9%	0	0.0%
Gynaecology	8	2.9%	8	2.5%	9	3.6%
Other	25	9.2%	22	6.9%	22	8.9%
EAES role						
None / Not applicable	239	87.5%	283	89.3%	216	87.4%
Executive board	8	2.9%	3	0.9%	6	2.4%
Technology committee	8	2.9%	6	1.9%	6	2.4%
Education committee	7	2.6%	12	3.8%	10	4.0%
Research committee	11	4.0%	13	4.1%	9	3.6%

### OR training

Overall, 76.7% (*n* = 206) of respondents indicated that OR-training was considerably affected during the pandemic with a 53.4% ± 22.7% decrease in training was reported. This was mainly due to cancellation of elective cases (80.7%, *n* = 167) and relocation of residents (42.0%, *n* = 87). Alternative training opportunities were introduced in 25.6% of respondents (*n* = 35), and compensated for 32.2% ± 17.9% of the missed training. Most often introduced alternative resources were webinars (77.4%, *n* = 41), e-learning modules / online courses (66.0%, *n* = 35), and videos of lectures/live surgery/tips and techniques/or others (52.8%, *n* = 28). In these cases, 77.4% (*n* = 41) of respondents had access at least once a week. Consensus was achieved on the importance of one statement ranking the preferred method among ten resources (Table 2a). Because only one statement achieved consensus, ranking was not performed. When asked how often residents should have access, 92% (*n* = 205) of all respondents agreed that residents should have access between once a day and once a week.

**Table 2:** Ranked importance of statements and resources

Statement. It is important that:		Consensus (%)	Mean rank
a. OR training			
1	Alternative resources are used to compensate for missed OR training experiences	83.51	not applicable
Resources			
1	Videos (live surgery)	91.48	4.46
2	Videos (Lectures / pre-recorded surgery / tips and techniques)	90.15	4.75
3	Wet lab training	83.22	4.86
4	Simulators	81.04	5.14
5	Dry lab training	78.17	5.31
6	Virtual reality skills trainers	73.15	5.89
7	Online courses	77.03	5.94
8	Digitally augmented box trainers	70.57	6.12
9	Webinars	75.53	6.18
10	E-learning modules	70.00	6.34
b. Wet lab and dry lab training			
1	Residents are trained in a wet lab	83.40	1.81
2	Residents are trained in a dry lab	80.00	2.05
3	Alternative resources are used to compensate for missed wet lab and/or dry lab training experiences	75.47	2.13
Resources			
1	Videos (live surgery)	87.88	2.50
2	Simulators	84.91	3.05
3	Videos (Lectures / pre-recorded surgery / tips and techniques)	84.09	3.26
4	Virtual reality skills trainers	77.27	4.12
5	Online courses	74.15	4.58
6	Digitally augmented box trainers	73.58	5.04
7	Webinars	70.72	5.44
c. Assessment, certification and accreditation			
1	Alternative resources are used to compensate for missed technical skills assessments during COVID	86.04	1.27
2	Alternative resources are used to assess technical skills assessments after COVID	81.39	1.73
Resources (consensus)			
1	Assessment in wet lab	76.36	1.45
2	Case discussion	76.97	1.55
Resources (60–70% agreement)			
1	Tele-assessment of skills using a live video	68.48	2.28
2	Assessment on simulator	67.45	2.41
3	Assessment on VR trainer	65.12	3.06
4	Tele-assessment of skills using a recorded video	61.12	3.38
5	Assessment on digital box trainer	61.09	3.86
d. Digital resources			
1	Feedback of learners is used to ensure quality control of digital resources	86.08	2.38
2	Digital resources are used to compensate for limited OR training experiences	79.33	2.45
3	Accreditation of digital resources is carried out by in-depth analysis by specialists	76.37	3.63
4	Quality assurance of digital resources is undertaken by training bodies or (inter)national societies	75.95	4.12
5	Digital resources are used to compensate for limited technical skills assessments	73.42	4.39
6	Digital resources are officially accredited by training bodies or (inter)national societies	73.00	5.36
7	Digital resources are used to compensate for limited wet lab and/or dry lab experiences	70.47	5.67
Resources			
1	Virtual reality trainers	75.51	2.33
2	Digitally augmented box trainers	74.48	2.56
3	E-learning modules / online courses	80.14	2.92
4	Webinars	81.56	3.14
5	Websites	77.44	4.06

Component		Consensus (%)	Mean rank
e. Webinar components			
1	Hands-on activity	83.09	3.65
2	Live videos of surgery	85.81	3.87
3	Tips and tricks	93.57	4.46
4	Pre-recorded surgical videos	88.73	4.66
5	Interactive case discussion	90.71	5.02
6	State-of-the art scientific information or interventions	87.05	5.54
7	Lectures	84.28	6.45
8	Expert opinions	89.29	6.50
9	Q&A	85.71	7.27
10	National or international accreditation	80.00	7.58

### Wet lab and dry lab training

Only 37.3% (*n* = 95) of residents had access to a wet and/or dry lab, and this access was disrupted in 43.2% (*n* = 41) of those respondents during the pandemic. The percentage of affected training time varied widely with a mean of 35.6% ± 33.8%, most often due to restricted access of residents (46.3%, *n* = 44). Alternative training opportunities were rarely introduced (14.7%, *n* = 14), and in these cases, those alternatives compensated for a mean of 43.2% ± 20.4% of missed training opportunities. The top most introduced alternatives were e-learning modules and online courses (10.5%, *n* = 10), webinars (10.5%, *n* = 10), websites (7.4%, *n* = 7), and videos of lectures, live surgeries, tips and techniques (7.4%, *n* = 7). Residents had access to these resources between once a day and once a week in 76.92% (*n* = 10). Consensus was achieved on the importance of three statements and seven resources (Table 2b).

### Assessment, certification, and accreditation

A total of 55.8% (*n* = 134) of respondents indicated that 55.6% ± 22.5% of the assessment of surgical skills has been affected since the pandemic. This was usually due to the impact of the pandemic on cancellation of elective cases (73.1%, *n* = 98) and relocation of residents (47.0%, *n* = 63). Alternative assessment methods were introduced in 17.9% (*n* = 24) of respondents, by using physical alternatives such as simulators (62.5%, *n* = 15) and tele-assessment (41.7%, *n* = 10). These methods compensated for 38.8% ± 21.1% of the affected skills assessments. Within the group of respondents who indicated that skills assessment were affected during the pandemic, face-to face assessment methods were still preferred (64.9%, *n* = 87) over remote assessments (28.4%, *n* = 38). Consensus was reached on alternative resources which required physical attendance (Table 2c). When respondents were asked to rank the remote resources on which 60–70% consensus was achieved, tele-assessment using live video and assessments on simulator were the highest ranking resources.

### Digital resources

**Fig. 1:**
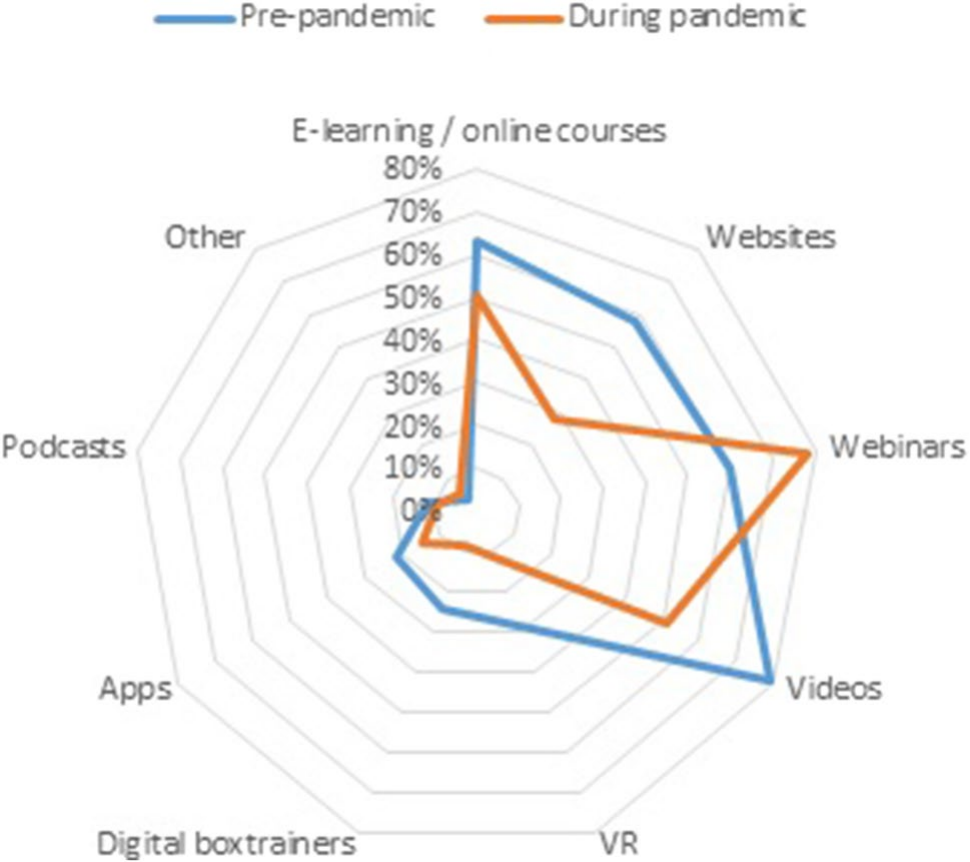
Overview of used digital resources before and since the pandemic

**Fig. 2:**
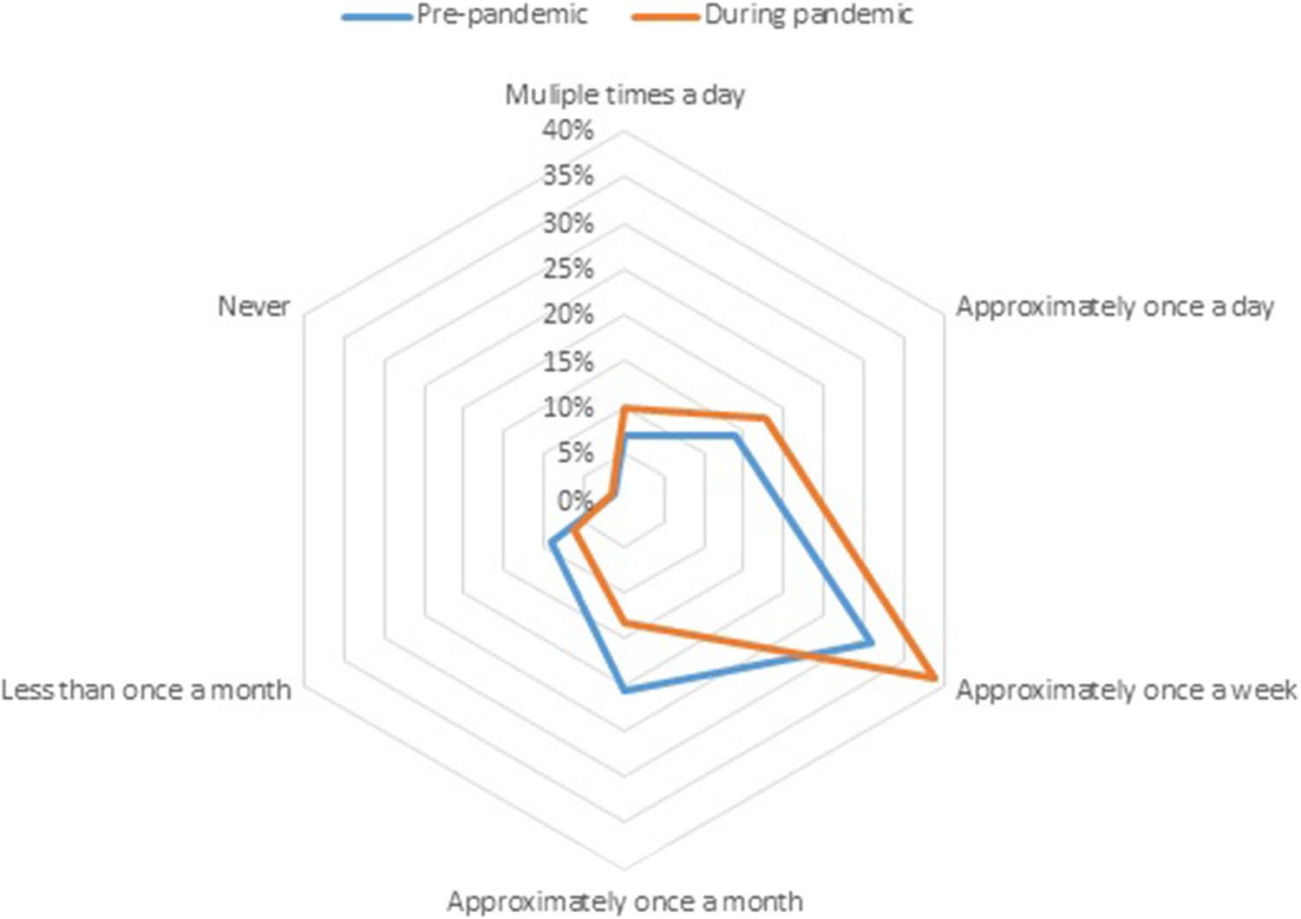
Overview of frequency of use of digital resources before and since the pandemic

Before the pandemic, digital resources had been previously incorporated in 43.5% (*n* = 93) of respondents and (new) resources were introduced during the pandemic in 45.3% (*n* = 97) of respondents–as indicated by surgeons and residents. When comparing which resources were introduced before or during the pandemic, webinars were most often implemented–although e-learnings, online courses, and the use of videos remained popular (Fig. 1). At the same time, respondents indicated that digital resources were used more often since the pandemic (Fig. 2d). Respondents achieved consensus on the importance of five resources and seven statement. Ninety percent of respondents believed that residents should have access to these resources at least once a month, and 70% believed that residents should have access for at least once a week.

Because there was an evident interest in webinars in the first survey of this Delphi, an additional item was added to the surveys on webinar components. Respondents achieved consensus on the importance of ten components of which Hands-on activity, live videos of surgery, and Tips & Tricks were ranked highest (Table 2e).

### Recommendations and overview of supported resources

Thirteen recommendations were formulated based on the statements on which consensus was achieved by the participants (Table 3). Three recommendations (1, 4, and 5) relate to the educational response to the pandemic alone; alternative resources should be used to compensate for missed OR training, wet lab and dry lab training, and assessments and accreditation. Additionally, alternative resources should be used to assess technical skills assessments after COVID (recommendation 6). Two recommendations relate to the importance of wet lab and dry lab training (2, 3), four relate to the quality control and accreditation of digital resources (7, 9, 10, 12), and three relate to the importance of using digital resources as compensation for limited training opportunities (8, 11, 13).

**Table 3:** Overview of recommendations based on statements on which consensus was achieved

Component	Recommendations	Resources
OR training	1. Alternative resources should be used to compensate for missed OR training experiences	1. Videos (live surgery)
		2. Videos (Lectures / pre-recorded surgery / tips and techniques)
		3. Wet lab training
		4. Simulators
		5. Dry lab training
		6. Virtual reality skills trainers
		7. Online courses
		8. Digitally augmented box trainers
		9. Webinars
		10. E-learning modules
Wet lab and dry lab training		1. Videos (live surgery)
		2. Simulators
	2. Residents should be trained in a wet lab	3. Videos (Lectures / pre-recorded surgery / tips and techniques)
	3. Residents should be trained in a dry lab	
	4. Alternative resources should be used to compensate for missed wet lab and/or dry lab training experiences	4. Virtual reality skills trainers
		5. Online courses
		6. Digitally augmented box trainers
		7. Webinars
Assessment, certification, and accreditation		1. Assessment in wet lab
		2. Case discussion
	5. Alternative resources should be used to compensate for missed technical skills assessments during COVID	3. Tele-assessment of skills using a live video *
	6. Alternative resources should be used to assess technical skills assessments after COVID	4. Assessment on simulator *
		5. Assessment on VR trainer *
		6. Tele-assessment of skills using a recorded video *
		7. Assessment on digital box trainer *
Digital resources		1. Virtual reality trainers
		2. Digitally augmented box trainers
	7. Feedback of learners should be used to ensure quality control of digital resources	3. E-learning modules / online courses
		4. Webinars
	8. Digital resources should be used to compensate for limited OR training experiences	5. Websites
	9. Accreditation of digital resources should be carried out by in-depth analysis by specialists	Webinar components
		1. Hands-on activity
	10. Quality assurance of digital resources should be undertaken by training bodies or (inter)national societies	2. Live videos of surgery
		3. Tips and tricks
	11. Digital resources should be used to compensate for limited technical skills assessments	4. Pre-recorded surgical videos
		5. Interactive case discussion
	12. Digital resources should be officially accredited by training bodies or (inter)national societies	6. State-of-the art scientific information or interventions
	13. Digital resources should be used to compensate for limited wet lab and/or dry lab experiences	7. Lectures
		8. Expert opinions
		9. Q&A
		10. National or international accreditation

*Agreement of 60–70%, consensus of ≥ 70% not achieved

## Discussion

The current web-based modified Delphi study establishes that the negative effects of the COVID-19 pandemic on surgical training were indeed perceived as severe within the EAES membership. Clinical training and assessment were reportedly > 50% affected, while wet lab and dry lab access were affected in over 35%. While participants achieved consensus on the urgency to use alternative resources as response to the pandemic and afterwards, alternatives that were introduced only compensated for 32.2–43.2% of missed training—if they were introduced at all. Video-based and hands-on training modalities such as wet labs, dry labs, and (VR) simulators were the preferred resources for all types of missed training, although only 37.3% of respondents had access to dry or wet labs.

Video-based training resources have long been of interest for surgeons, and were the go-to resource of educators during the pandemic [8–10]. Participants in the current study preferred live videos over pre-recorded videos of surgery, indicating their preference to replicate the experience that residents have while standing at the table in the OR. Several surgical livestreams were successfully initiated during the pandemic, although there are some ethical, safety, and medico-legal considerations: Informed consent is critical, patient safety can never be compromised to improve the educational value videos, and recording of videos by others should be prevented [8, 11–13]. It is easier and recommended to safeguard these challenges in pre-recorded videos, although in the current study live videos were better appreciated. The extensive online availability of pre-recorded videos is another advantage. Sites such as YouTube, WebSurg, and Advances in Surgery (AIS) offer a wide variety of surgical interventions and don’t require any time, effort or equipment to use. Before deciding to record and transmit live videos of surgery, it is therefore prudent to determine if a video of the intended intervention is already available and free to use. A possible augmentation can be to discuss the video and thereby providing an interactive learning experience.

Hands-on training resources, i.e. wet labs and dry labs, provide valuable training opportunities for surgical residents outside of the clinic and have been validated across a range of surgical specialties[14–17]. Unsurprisingly, respondents in the current study reached consensus on the need to use these resources both for their own merit, and as compensation for missed/limited OR training. Surprisingly, only 37.3% of respondents indicated that they had access to wet or dry labs. There is no clear reason for the lack of access to these facilities—no scientific or grey literature is available on the lack of wet and dry labs—although financial and organizational factors probably play a role. Solving the discrepancy in proven value and preference of respondents on one side, and the lack of access to these facilities on the other, seems vital and can come in multiple forms. One option is to arrange financial and organizational support for national or local initiatives to establish more wet and/or dry labs to reduced variability in training resources and opportunities. Another option would be to use and invest in digital skills trainers–such as the VR simulators, which were the best ranked digital resource in this study. There are five recent RCT’s available which compare the outcomes of wet/dry labs and VR trainers [18–22]. These demonstrate that while VR may be promising, it is not sophisticated enough to replace training hands-on in a facility. The wet/dry lab facilities significantly outperformed the group which practices with a VR trainer in four studies, and performances were equal in only one study [22]. Endorsing curricula and quality control and accreditation of digital resources by (inter)national surgical bodies such as the EAES can be a start in improving the value of these resources in surgical training, as reached consensus on in this study.

Although participants achieved consensus on the need to use digital resources when training is limited, digital resources were currently seldom used. Digital resources were never in the top three ranked resources for OR, wet lab and dry lab training, and not among the resources on which a consensus was achieved for assessment, certification, and accreditation. In addition, even if digital resources are available, it is uncertain if are used by residents in their day-to-day training [23]. There are therefore several organizational factors to consider when implementing and adopting innovative educational resources. While there are no studies available which evaluate these factors specifically for digital educational resources, eHealth initiatives are fortunately better investigated. Although digital education and training resources are considerably different from eHealth initiatives, the adoption, implementation and scale up of new technologies will presumably follow a similar path [24–26]. Støme et al. analysed 27 articles reporting on adoptions of eHealth solutions, and identified data management, user adaptations, and evaluation and scaling as the most important factors that enable adoption [26]. A study by Gijsbers et al. identified six themes affecting upscaling of telemonitoring; norms & attitudes, organizational structure & process, resources, policies and incentives, network & linkages, and media & change agents [27]. To safeguard the use of resources by residents it is important to implement the resource in a curriculum and provide sufficient time to train [28, 29].

Using a modified Delphi approach, this study aims to evaluate the impact of COVID on surgical resident training and to reach consensus on how best to response to the pandemic. The strength of the study lies in (a) the robustness of the Delphi methodology, (b) the population of interest (complete EAES membership was approached) and (c) the large number of participants responding throughout all three survey rounds. Because the Delphi methodology is based on the assumption that all participants are more or less equal in skills, knowledge and experience, the effects of COVID-19 on surgical practice may have confounded results which can be seen as a limitation of this study. This confounding effect was limited by the over-all high number of respondents and the fact that the responses proved to be a demographically representative sample across the EAES-membership. Unfortunately this representativeness also results in few participating residents and unclarity with regard to the educational background of participants. While the latter was compensated to some extent by the educational expertise of the senior authors of this manuscript, further efforts involving these groups are needed to strengthen the results of this study.

## Conclusions

This study highlights the severe international impact of the COVID pandemic on MIS–training and reports on agreed statements of recommendation with regard to OR training, wet lab and dry lab training, assessment, certification, and accreditation, as well as digital resources and simulation to support surgical training during the recovery from the pandemic. Face-to-face hands-on training is still the preferred learning method, although respondents indicated that digital and remote training are valuable additions to the training palette. While surgical educators are resolving the challenges met during the COVID pandemic, and (inter)national training bodies have issued statements and guidelines on this topic, the current study can support and guide training curricula to compensate for the training gaps that are generated by the pandemic. Organizations such as the EAES are encouraged to support surgical educators in using and implementing these resources in a suitable and sustainable way. Insights from this Delphi can further help (inter)national governing training bodies and hospitals in futureproofing resident curricula in post-pandemic times.
